# Diagnostic Value of Fecal Lactoferrin in Differentiating Inflammatory Bowel Disease From Irritable Bowel Syndrome: A Comparative Study in a Bangladeshi Cohort

**DOI:** 10.7759/cureus.82187

**Published:** 2025-04-13

**Authors:** AKM Jubaere, Fazlul Karim Chowdhury, Abdullah Al Shah Newaz, Susmita Islam, Shah Alam Miah, Mohammad Shoaib Chowdhury, Sumona Islam

**Affiliations:** 1 Department of Gastroenterology, Bangabandhu Sheikh Mujib Medical University, Dhaka, BGD; 2 Department of Gastroenterology, Shaheed Syed Nazrul Islam Medical College, Dhaka, BGD

**Keywords:** crohn's disease, crohns disease, fecal lactoferin, fecal lactoferrin, inflammatory bowel disease, inflammatory bowel disease, irritable bowel syndrome, ulcerative colitis, ulcerative colıtıs

## Abstract

Background

Inflammatory bowel disease (IBD) and irritable bowel syndrome (IBS) share overlapping symptoms, making differentiation challenging. IBD, which includes ulcerative colitis (UC) and Crohn’s disease (CD), involves chronic inflammation requiring long-term management, whereas IBS is a functional gastrointestinal disorder without organic pathology. Fecal lactoferrin, a biomarker of intestinal inflammation, has emerged as a potential noninvasive diagnostic tool for distinguishing IBD from IBS.

Objective

This study aimed to measure and compare fecal lactoferrin levels in patients with IBD, IBS, and healthy controls to evaluate its diagnostic accuracy in differentiating IBD from IBS in a resource-limited setting.

Methods

A cross-sectional comparative study was conducted at the Department of Gastroenterology, Bangabandhu Sheikh Mujib Medical University (BSMMU), Dhaka, Bangladesh, from January 2016 to June 2017. The study included 50 diagnosed IBD patients, 25 IBS patients (based on Rome III criteria), and 25 healthy controls. Fecal lactoferrin levels were measured using enzyme-linked immunosorbent assay (ELISA), with a cutoff value of 7.75 µg/g considered indicative of intestinal inflammation. Sensitivity, specificity, positive predictive value (PPV), and negative predictive value (NPV) were calculated. Data were analyzed using IBM SPSS Statistics for Windows, Version 25 (Released 2017; IBM Corp., Armonk, New York, United States), with statistical significance set at p < 0.05.

Results

The mean fecal lactoferrin levels were significantly higher in IBD patients (83.01 ± 48.54 µg/g) compared to IBS patients (6.39 ± 2.69 µg/g) and healthy controls (6.80 ± 2.30 µg/g) (p < 0.001). At a cutoff of 7.75 µg/g, fecal lactoferrin demonstrated 88% sensitivity, 84% specificity, 92% PPV, and 78% NPV in distinguishing IBD from IBS. The area under the receiver operating characteristic (ROC) curve was 0.908, indicating excellent diagnostic performance.

Conclusion

Fecal lactoferrin is a highly sensitive and specific biomarker for differentiating IBD from IBS. Its use as a noninvasive diagnostic tool could reduce unnecessary endoscopic procedures and aid in early and accurate diagnosis, particularly in resource-limited settings like Bangladesh. Further large-scale studies are recommended to validate these findings.

## Introduction

Inflammatory bowel disease (IBD) and irritable bowel syndrome (IBS) are prevalent gastrointestinal disorders that share overlapping clinical manifestations, such as abdominal pain, diarrhea, and altered bowel habits. However, they differ significantly in terms of pathophysiology, prognosis, and therapeutic approaches [1,2]. IBD encompasses chronic inflammatory conditions of the gastrointestinal tract, primarily ulcerative colitis (UC) and Crohn’s disease (CD), both of which require long-term medical care and may lead to serious complications [3]. In contrast, IBS is a functional gastrointestinal disorder characterized by recurrent abdominal discomfort and altered bowel habits in the absence of organic pathology, with most patients experiencing a favorable prognosis [4].

The global prevalence of IBD has been increasing, particularly in Western countries, with peak incidence occurring between the second and fourth decades of life. Genetic predisposition and environmental factors such as smoking, diet, infections, and socioeconomic conditions play significant roles in disease development [3,5]. In Bangladesh, the prevalence of IBD is lower than in Western nations, yet its diagnosis remains a challenge due to limited access to advanced diagnostic tools [6]. On the other hand, IBS is more common, with studies reporting a prevalence of 20.6% in men and 27.7% in women in Bangladesh [7]. The condition significantly impacts quality of life, leading to increased healthcare utilization and economic burden [7,8].

Despite the availability of diagnostic criteria such as the Rome III criteria for IBS, distinguishing between IBD and IBS based solely on symptoms remains difficult, especially in the absence of rectal bleeding and systemic illness [9]. Physicians often rely on invasive investigations such as colonoscopy and radiographic imaging to rule out IBD, which can expose patients to procedural risks and financial strain. A considerable number of patients with functional gastrointestinal disorders undergo unnecessary endoscopic evaluations, while some IBD cases remain undiagnosed for extended periods due to misclassification as IBS [3,5].

An ideal diagnostic tool should be cost-effective, noninvasive, and reliable in differentiating IBD from IBS. Fecal biomarkers, particularly fecal lactoferrin, have emerged as promising diagnostic tools. Lactoferrin is an iron-binding glycoprotein secreted by neutrophils and mucosal epithelial cells, serving as a marker of intestinal inflammation. Elevated fecal lactoferrin levels are associated with active IBD, whereas IBS patients typically exhibit normal levels. Studies have demonstrated that fecal lactoferrin testing offers high sensitivity and specificity for detecting intestinal inflammation, making it a valuable adjunct in clinical decision-making [10,11].

In Bangladesh, where healthcare resources are limited, incorporating fecal lactoferrin testing into routine clinical practice could aid in early and accurate differentiation of IBD from IBS, reducing unnecessary endoscopic procedures and improving patient management. The present study aims to measure and compare fecal lactoferrin levels in patients with IBD and IBS, providing insights into its potential role as a noninvasive screening tool in the local healthcare setting.

## Materials and methods

Study design and setting

This cross-sectional comparative study was conducted in the Department of Gastroenterology, Bangabandhu Sheikh Mujib Medical University (BSMMU), Dhaka, Bangladesh, from January 2016 to June 2017.

Study population and patient selection

The study included diagnosed cases of IBD and IBS, as well as healthy controls. IBD diagnosis was based on a combination of clinical history, physical examination, laboratory findings, imaging, and colonoscopy with biopsy confirmation. IBS was diagnosed according to the Rome III criteria after ruling out organic causes.

Inclusion and exclusion criteria

For IBD patients, the inclusion criteria included age above 18 years and a confirmed diagnosis of IBD. Exclusion criteria included indeterminate colitis, microscopic colitis, infectious colitis, intestinal tuberculosis, intestinal lymphoma, ischemic enteritis or colitis, radiation enteritis or colitis, and drug-induced enteritis. For IBS patients, the inclusion criteria included age above 18 years and meeting the Rome III criteria. Exclusion criteria included alarm features (anemia, fever, weight loss, per rectal bleeding, family history of colon cancer), organic bowel disease, concurrent illnesses explaining gastrointestinal symptoms (e.g., thyroid disease, diabetes), history of major gastrointestinal surgery (except appendectomy), and pregnancy. Healthy controls were individuals who had no gut-related symptoms and were apparently healthy.

Sample size calculation

The sample size was determined using power analysis for a single proportion. Based on previous studies, a sensitivity of 95% for fecal lactoferrin in identifying IBD was hypothesized [12]. The final sample size included 50 IBD patients, 25 IBS patients, and 25 healthy controls.

Data collection and study procedure

All relevant patient data were recorded using predesigned data collection sheets. Stool samples were collected from 100 participants, including 50 with confirmed IBD, 25 with IBS, and 25 healthy controls. Laboratory analyses were performed in the Department of Laboratory Medicine, BSMMU, using the lactoferrin enzyme-linked immunosorbent assay (ELISA) kit. A cutoff level of >7.25 mcg/g of stool was considered positive for inflammation.

Specimen collection and laboratory analysis

Stool samples (minimum 0.3 g) were collected in sterile containers without preservatives and stored appropriately at -20°C or -80°C to preserve bioactivity. The ELISA test was conducted following the manufacturer's guidelines, measuring absorbance at 450 nm to determine lactoferrin concentration.

Data analysis

Data were analyzed using IBM SPSS Statistics for Windows, Version 25 (Released 2017; IBM Corp., Armonk, New York, United States). Results were presented using frequency distributions and percentages. A p-value less than 0.05 was considered statistically significant. Sensitivity and specificity were calculated with 95% confidence intervals. Receiver operating characteristic (ROC) curves were generated to determine the optimal cutoff for disease identification.

Ethical considerations and confidentiality

Ethical approval was obtained from the Institutional Review Board (IRB) of Bangabandhu Sheikh Mujib Medical University under approval number 11636, and all participants provided informed written consent. Patient information and test results were kept confidential, with access restricted to the research team and regulatory authorities.

## Results

This study enrolled a total of 100 participants, including 50 patients with IBD, 25 patients with IBS, and 25 healthy controls. Colonoscopy and biopsy were performed in both IBD and IBS patients. Fecal lactoferrin levels were measured in all groups. The demographic characteristics of the study population are summarized in Table 1.

**Table 1:** Demographic characteristics IBD: inflammatory bowel disease; IBS: irritable bowel syndrome

Variables	Variables	IBD	IBS	Control	p-value
Age		35.8	32.40	53.7	0.173
Gender	Male	33 (66%)	17 (68%)	20 (80%)	0.18
	Female	17 (34%)	8 (32%)	5 (20%)	
Residence	Rural	25 (50%)	10 (40%)	8 (32%)	0.16
	Urban	15 (30%)	9 (36%)	10 (40%)	
	Semi urban	10 (20%)	6 (24%)	7 (28%)	
Smoking status	Smoker	16 (32%)	8 (32%)	6 (24%)	0.7
	Past smoker	9 (60%)	9 (36%)	10 (40%)	
	Non-smoker	25 (50%)	8 (32%)	9 (36%)	

Hemoglobin levels were significantly lower in the IBD group compared to the IBS group, while erythrocyte sedimentation rate (ESR) and C-reactive protein (CRP) were significantly higher in the IBD group than in the IBS group. The details are presented in Table 2.

**Table 2:** Laboratory investigations in IBD and IBS group Data are presented as mean ± standard deviation Hb: hemoglobin; ESR: erythrocyte sedimentation rate; CRP: C-reactive protein; IBD: inflammatory bowel disease; IBS: irritable bowel syndrome

Variables	IBD	IBS	p-value
Hb (gm/dl)	10.86 ± 0.58	11.74 ± 0.65	<0.001
ESR (1st hour)	49.26 ± 14.35	30.96 ± 6.13	<0.001
CRP (mg/l)	14.13 ± 8.47	4.37 ± 0.93	<0.001

Tables 3-4 show that the mean fecal lactoferrin levels (µg/g of stool) were 83.01 ± 48.54 in IBD patients, 6.39 ± 2.69 in IBS patients, and 6.80 ± 2.30 in the healthy control group. Among IBD subtypes, the mean fecal lactoferrin level was 77.47 ± 35.91 in CD and 85.85 ± 52.72 in UC. The fecal lactoferrin levels in IBD patients were significantly higher compared to both IBS patients and the healthy control group, highlighting its potential as a biomarker for distinguishing IBD from non-inflammatory conditions.

**Table 3:** Fecal lactoferrin level in IBD, IBS, and control groups Data are presented as mean ± standard deviation IBD: inflammatory bowel disease; IBS: irritable bowel syndrome

Variables	IBD	IBS	Control	p-value
Fecal lactoferrin (µg/g of stool)	83.01 ± 48.54	6.39 ± 2.69	6.80 ± 2.30	<0.001

**Table 4:** Fecal lactoferrin level in CD and UC Data are presented as mean ± standard deviation CD: Crohn's disease; UC: ulcerative colitis

Variables	CD	UC	p-value
Fecal lactoferrin (µg/g of stool)	77.47 ± 35.91	85.85 ± 52.72	<0.001

Table 5 shows the sensitivity, specificity, positive predictive values (PPVs), negative predictive values (NPVs), and accuracy at different cut-off values, ranging from the lowest (6.5 µg/gm) and 7.75 µg/gm to the highest (10.0 µg/gm) of stool.

**Table 5:** Performance of diagnostic tests of level of fecal lactoferrin at different cut-off values PPV: positive predictive value; NPV: negative predictive value

Cut-off values (7.75 µg/gm stool)	Sensitivity	Specificity	PPV	NPV
6.5	90%	48%	78%	44%
7.75	88%	84%	92%	78%
10	86%	88%	93%	75%

Table 6 shows fecal lactoferrin levels in different groups. Among 50 IBD patients, 44 had levels above 7.75 µg/g, while six had levels below 7.75 µg/g. In the IBS group, four patients had levels above 7.75 µg/g, and 21 had levels below this threshold. In the healthy control group, three individuals had levels above 7.75 µg/g, while 22 had levels below. The difference in fecal lactoferrin levels between the IBD and IBS groups was statistically significant (p < 0.001).

**Table 6:** Distribution of the study population according to groups with a level of fecal lactoferrin at a cut-off value of 7.75 µg/g of stool IBD: inflammatory bowel disease; IBS: irritable bowel syndrome

Fecal lactoferrin (µg/g of stool)	IBD	IBS	Controls	p-value
>7.75	44 (88%)	4 (16%)	3 (12%)	<0.001
≤7.75	6 (12%)	21 (84%)	22 (88%)	

**Figure 1:**
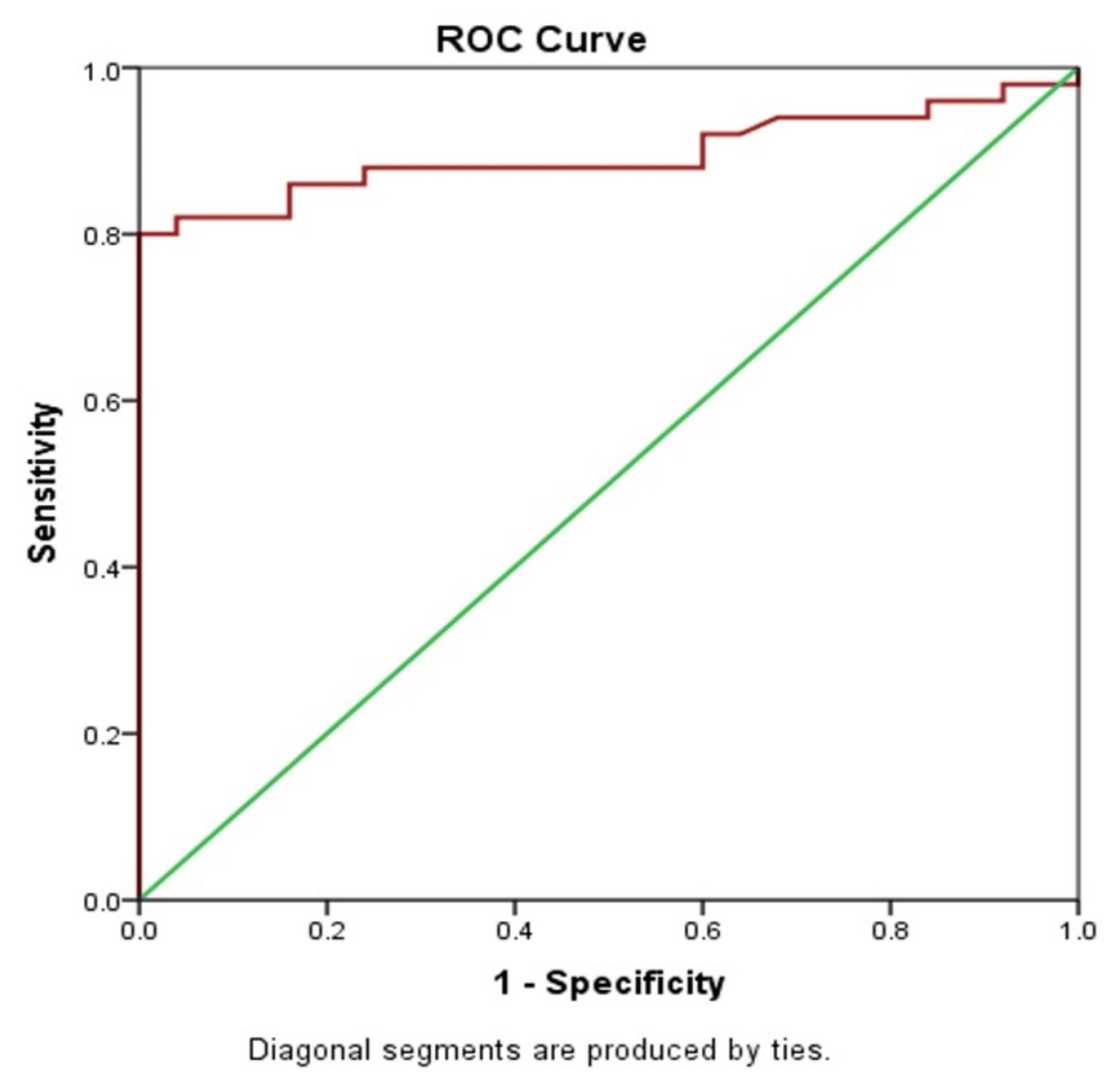
ROC of fecal lactoferrin for detecting intestinal inflammation ROC: receiver operating characteristic

The ROC curve was generated by plotting the true positive rate against the false positive rate as the classification threshold increased from 0 to 1 (Figure 1).

A highly effective classifier exhibits a rapid rise in the true positive rate, resulting in an area under the curve (AUC) close to 1. In this study, the classifier achieved an AUC score of 0.908, indicating excellent performance. Moreover, the difference in test results between the two groups was highly significant (p < 0.001).

## Discussion

This comparative study was conducted in the Department of Gastroenterology, BSMMU, in collaboration with the Department of Laboratory Medicine, BSMMU. The primary objectives were to measure fecal lactoferrin levels in patients with IBD and IBS and compare these values between the two groups. A total of 100 patients were included, comprising 50 IBD patients, 25 IBS patients, and 25 healthy controls. The study population was recruited from both the inpatient and outpatient departments of gastroenterology, BSMMU.

In this study, quantitative estimation of fecal lactoferrin was performed across all three groups using the same method as previous studies by Walker et al., Sidhu et al., and Kane et al. [10,12,13]. Our results align with these studies, demonstrating significantly elevated fecal lactoferrin levels in IBD patients compared to IBS and healthy controls, reinforcing its utility as a biomarker for distinguishing inflammatory and non-inflammatory bowel conditions [10,12,13].

Age and gender distribution

The mean age of IBD patients in our study was 35.80 years, while for IBS patients, it was 32.40 years. These values were slightly higher than those reported by Molodecky et al., who found that IBD most commonly presents between 20 to 30 years of age [5]. The observed difference in our study may be due to delayed diagnosis, limited access to healthcare, and a lack of awareness about early symptoms, leading to late presentation. Similarly, Sidhu et al. in the UK reported a higher mean age for IBD (42 years) and IBS (58 years) than in our cohort [13]. The variation in mean age across studies may be influenced by differences in patient selection criteria, such as age range inclusion (18-70 years in our study) and the higher prevalence of IBD in elderly populations in Western countries [3].

Regarding gender distribution, we observed that both IBD and IBS were more prevalent in males in our study. Among IBD patients, males constituted a higher proportion than females, aligning with some previous studies but differing from others that reported a female predominance in CD [12,13]. Similarly, in the IBS group, males (68%) outnumbered females (32%), which contrasts with Hildebrand et al., who reported a female predominance in IBS [14]. This discrepancy may be attributed to our small sample size (n=25) and potential differences in healthcare access, where men in our region may be more likely to seek medical care than women [6].

Fecal lactoferrin levels in IBD, IBS, and healthy controls

Our study found that fecal lactoferrin levels were significantly higher in IBD patients (83.01 ± 48.54 µg/g) than in IBS patients (6.39 ± 2.69 µg/g) and healthy controls (6.80 ± 2.30 µg/g). These results are in line with previous studies.

Buderus et al. reported much higher fecal lactoferrin levels in IBD patients (313.60 ± 46.4 µg/g in CD and 370.70 ± 46.90 µg/g in UC), while IBS patients had extremely low levels (1.3 ± 0.5 µg/g) [15].

Walker et al. found even more striking differences, with fecal lactoferrin levels reaching 1880 ± 565 µg/g in UC, 1701 ± 382 µg/g in CD, and just 1.17 ± 0.47 µg/g in IBS [10].

Compared to these studies, our IBD patients had lower fecal lactoferrin levels. This difference can be explained by the disease status at the time of sample collection. Most of our IBD patients were already on long-term treatment, whereas in studies like Walker et al. and Buderus et al., lactoferrin levels were measured before treatment initiation in newly diagnosed patients with active disease [10,15]. This highlights the importance of considering disease activity and treatment status when interpreting fecal lactoferrin results.

Another limitation of our study was the inability to categorize IBD patients into active and inactive disease groups using indices such as the Harvey-Bradshaw Index for CD or the Mayo Score for UC. This was due to time constraints and a lack of follow-up assessments, which may have influenced the overall mean fecal lactoferrin values.

Diagnostic performance of fecal lactoferrin

To evaluate the diagnostic accuracy of fecal lactoferrin in differentiating IBD from IBS, we performed ROC curve analysis, yielding an AUC score of 0.908 (95% CI: 0.839-0.987). This high AUC value indicates that fecal lactoferrin is a very reliable marker for distinguishing between IBD and IBS (p < 0.001).

In our study, we conducted ROC analysis to determine the optimal cut-off values for fecal lactoferrin in differentiating IBD patients from healthy controls and those with IBS. We identified three potential cut-off values: 6.5 µg/g, 7.75 µg/g, and 10 µg/g. Among these, we selected 7.75 µg/g as the final cut-off value, as the majority of the patient values were concentrated around this threshold.

Other studies have used different cut-off values for fecal lactoferrin. For example, Kane et al. used 4 µg/g, while Sidhu et al. used >7.25 µg/g. Zhou et al. reported a sensitivity of 78% and specificity of 94% for fecal lactoferrin in diagnosing IBD, providing further context for our own findings [12,13,16].

At a cut-off value of 7.75 µg/g, our study showed a sensitivity of 88%, a specificity of 84%, a PPV of 92%, and a NPV of 78%. When we considered a higher cut-off of 10 µg/g, sensitivity slightly decreased to 86%, while specificity increased to 88%. At this threshold, the PPV rose to 93%, and the NPV dropped to 75%. These findings highlight the efficacy of fecal lactoferrin as a diagnostic marker for IBD, particularly at the 7.75 µg/g cut-off.

These findings demonstrate that higher cut-off values improve specificity but slightly reduce sensitivity, which aligns with prior studies by Kane et al. and Zhou et al. [12,16].

Fecal lactoferrin in IBS and healthy controls

Interestingly, four IBS patients had elevated fecal lactoferrin levels. This suggests that some IBS cases may have underlying low-grade inflammation that is not detectable by colonoscopy or histopathology. Similarly, three healthy individuals also showed elevated fecal lactoferrin levels, which might indicate subclinical inflammation despite the absence of symptoms.

Clinical implications

Fecal lactoferrin is an inexpensive, non-invasive marker that can assist clinicians in distinguishing between IBD and IBS, particularly in detecting active disease. Given its high sensitivity and specificity, it can be used to stratify patients who require further endoscopic evaluation, thereby reducing unnecessary procedures in IBS patients while ensuring early detection of IBD.

Limitations

Several limitations exist in this study. The sample size was relatively small, which may limit the generalizability of the findings to a broader population. Conducted at a single tertiary care center, the study may have introduced selection bias. The study also did not assess variations in fecal lactoferrin levels based on disease severity or treatment status, potentially impacting its diagnostic utility. Large-scale, multicenter studies with longitudinal follow-up are needed to validate these findings and further explore the role of fecal lactoferrin in distinguishing IBD from IBS.

## Conclusions

Our study demonstrates that fecal lactoferrin is a valuable biomarker for differentiating IBD from IBS and healthy controls. The significant elevation of fecal lactoferrin levels in IBD patients, as compared to those with IBS and healthy individuals, highlights its potential as a non-invasive, cost-effective diagnostic tool for IBD. The optimal cut-off value for fecal lactoferrin in our study was determined to be 7.75 µg/g, with high sensitivity, specificity, and predictive values, making it a reliable marker for identifying active disease. Despite some limitations, including the inability to categorize disease activity in IBD patients, the results support the use of fecal lactoferrin in clinical practice to help stratify patients and guide further diagnostic investigations. Future studies with larger sample sizes and a focus on disease activity categorization could further refine its clinical utility.
